# Supplementary material for: From Nuisance to Signal: Leveraging Close Relatives in Biobank-Scale Demographic Inference
Source: bioRxiv. 2026 Jun 19:2026.06.15.729614. Preprint. [Version 1] doi: 10.64898/2026.06.15.729614 (PMC13308010; doi:10.64898/2026.06.15.729614)
Supplement: Supplement 1 [file NIHPP2026.06.15.729614v1-supplement-1.pdf]

bioRxiv preprint doi: https://doi.org/10.64898/2026.06.15.729614; this version posted June 19, 2026. The copyright holder for this preprint (which was not certified by peer review) is the author/funder, who has granted bioRxiv a license to display the preprint in perpetuity. It is made available under a CC-BY 4.0 International license.

Supplementary Material

3.1 Methods

3.1.1 Creating Wright-Fisher Genealogies

Let $N_e[g]$ be the effective population size at generation g. We assume an equal number of males and females, such that each individual in generation $g-1$ picks a mother and father from $N_e[g]/2$ individuals. We use `msprime`'s `DemographyDebugger.population_size_trajectory` function to query $N_e[g]$, ensuring that the correct $N_e[g]$ is used. This is important for more complex demographies. Our pipeline supports both monogamous and non-monogamous models. In the monogamous model, if an individual picks a mother who already has an offspring assigned, then the father will not be randomly chosen and will be the father of that already-assigned offspring. In the non-monogamous model, mothers and fathers are chosen randomly and independently of each other.

3.1.2 Simulating with `msprime`

We first perform the WF simulation using the WF population pedigree generated in the step above. A demographic model is not provided, as the demography is (implicitly) encoded in the population pedigree. Once the simulation ends at $g = 25$ generations in the past, the result is a tree sequence, ts_1, with un-coalesced lineages. To complete the simulation, a new simulation is run with the desired demographic history, using ts_1 as the initial state and producing ts_2, in which all lineages are coalesced.

For the Quebec simulations, we start with ts_2 as provided by Anderson-Trocmé et al. [2]. We refer the reader to the original paper for details on these simulations. Briefly, they used `msprime`'s `fixed-pedigree` mode to simulate through the BALSAC genealogy [18, 29] to create the equivalent of ts_1 and then used an Out-of-Africa (European) demographic history to complete the simulation for the un-coalesced lineages in order to create ts_2. These simulations were run on 22 autosomes with realistic human recombination maps.

3.1.3 Creating realistic genotype data

We simulate genotype data using ts_2. First, we place mutations on the tree sequences (mutation rate 10^{-8}). Next, we downsample the sites in the tree sequence to achieve a marker density of approximately 600 SNPs/Mb ($\sim 1.8M$ SNPs genome-wide) in order to accurately call small IBD segments. Browning and Browning [4] found 50% power to detect 2 cM IBD segments with 550K SNPs, so the $\sim$ 3x SNP density we use should be sufficient.

We sampled sites from the minor allele frequency (MAF) histogram of the UK Biobank, using the following intervals: zero (fixed), (0, 0.01], (0.01, 0.05], (0.05, 0.1], and then four intervals of 0.1 width from 0.1 to 0.5. We then sampled, from the tree sequence, bi-allelic sites from these intervals to match the desired SNP density. These genotype data were then stored in a VCF for IBD calling.

bioRxiv preprint doi: https://doi.org/10.64898/2026.06.15.729614; this version posted June 19, 2026. The copyright holder for this preprint (which was not certified by peer review) is the author/funder, who has granted bioRxiv a license to display the preprint in perpetuity. It is made available under a CC-BY 4.0 International license.

3.1.4 Sampling IBD segments

For a pair of individuals (i, j), we denote the total length (in cM) of IBD segments they share $S_{i,j}$. If G is the total length of the haploid genome, then $k_{i,j} = \frac{S_{i,j}}{2G}$ denotes the proportion of IBD sharing for the pair. Now consider $t \in [0, 1]$ to be an IBD sharing proportion threshold. If $k_{i,j} > t$, the pair are considered "related" and if $k_{i,j} < t$, the pair are considered "unrelated". In this study, we use $t = 0.0883$, which represents a lower bound separating 3rd and 4th degree relatives [19].

We create a graph G using `networkx` in which individual i is represented as node n_i. An edge is added between nodes n_i and n_j if $k_{i,j} > t$. Our sampling schemes operate on this graph to produce four sampling conditions, each retaining a fraction f of the full cohort of N individuals, giving us ceil(Nf). We used $f = 0.25$, unless otherwise noted.

Filtering: related. To construct a subsample enriched for close relatives, we iterate through all pairs (i, j) with an edge in G in descending order of $k_{i,j}$. At each step, both individuals in the pair are added to the selected set. Iteration continues until the target size is reached or all related pairs are exhausted. If the number of individuals involved in related pairs falls short of the target, the remainder is filled by drawing uniformly at random (without replacement) from the unselected pool.

Filtering: unrelated. To construct a subsample purged of close relatives, we seek a large independent set of G. We process each connected component of G separately. Isolated nodes (singletons) are admitted directly. For each non-trivial component, we apply a greedy algorithm: at every iteration, all candidate nodes are sorted in ascending order of their current degree within the component, and the lowest-degree node is tentatively selected. The node is added to the output set only if it shares no edge with any previously selected node; the process then repeats until no further node can be added without violating independence, or the global target is reached. If the total number of selected nodes exceeds the target, a random subset of size Nf is drawn from them.

Filtering: random. As a baseline condition, Nf individuals are drawn uniformly at random without replacement from the full cohort, without regard to relatedness.

3.2 Software

3.2.1 `hap-ibd` [5]

`hap-ibd` was run with default parameters.

3.2.2 `IBDNe` [6]

We ran `IBDNe` with default parameters, including a minimum IBD segment length of 2 cM. We ran it with `filtersamples=True`, which removes 2nd degree relatives, and `filtersamples=False`.

3.2.3 `HapNe-IBD` [9]

`HapNe-IBD` was run with default parameters. We modified the HapNe-IBD code in order to support our 30 equal-length chromosome genome. The software normally takes as input the

bioRxiv preprint doi: https://doi.org/10.64898/2026.06.15.729614; this version posted June 19, 2026. The copyright holder for this preprint (which was not certified by peer review) is the author/funder, who has granted bioRxiv a license to display the preprint in perpetuity. It is made available under a CC-BY 4.0 International license.

genome build, which it uses to define chromosome arms. It then computes the IBD segment length spectra for each chromosome arm. We modified the code such that it works on 60 chromosome arms, each of 50 Mb.

3.3 Results

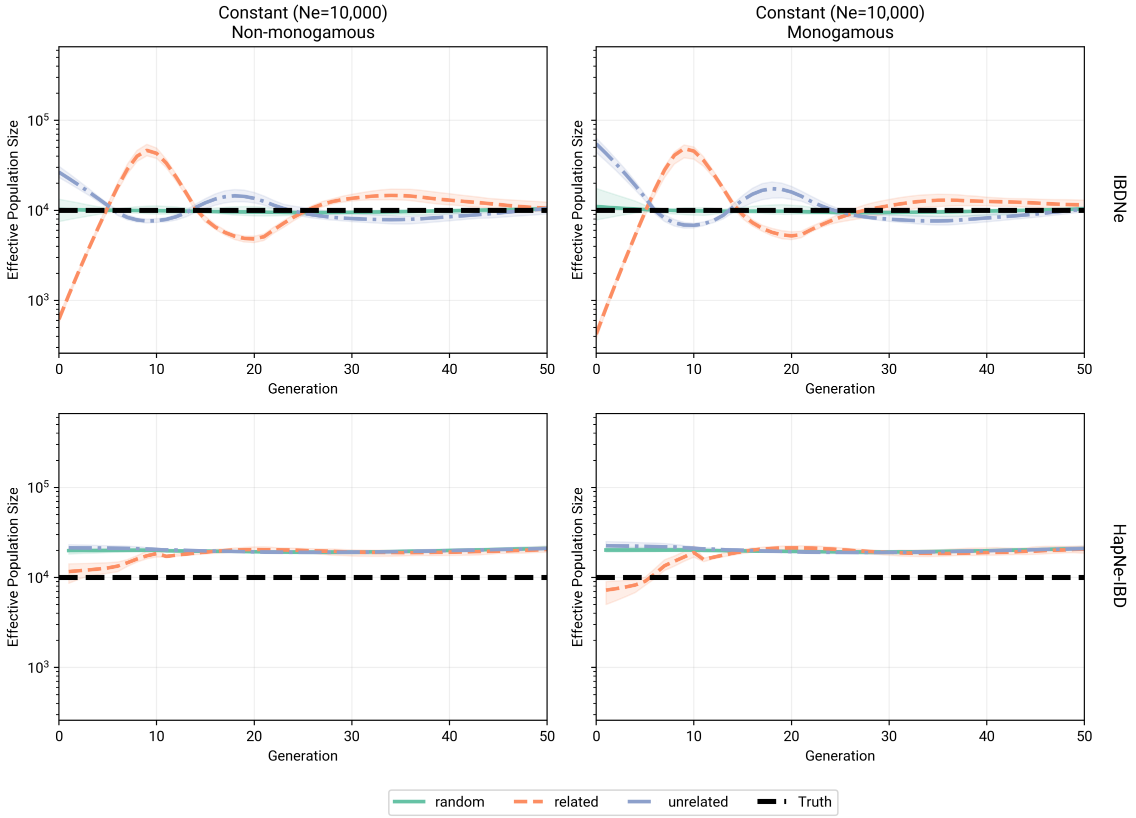

Figure 3: $N_e(t)$ inference for constant population size $N_e = 10,000$ for non-monogamous (left panels) and monogamous (right panels) mating schemes. Results for IBDNe (top panels) and HapNe-IBD (bottom panels) are shown. Qualitatively, we see no difference in $N_e(t)$ inference, in agreement with [32].

bioRxiv preprint doi: https://doi.org/10.64898/2026.06.15.729614; this version posted June 19, 2026. The copyright holder for this preprint (which was not certified by peer review) is the author/funder, who has granted bioRxiv a license to display the preprint in perpetuity. It is made available under a CC-BY 4.0 International license.

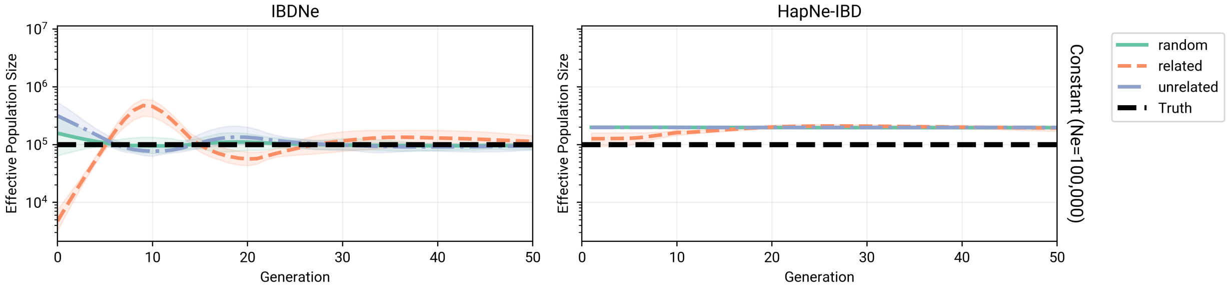

Figure 4: Inferred $N_e(t)$ for IBDNe and HapNe-IBD for a constant $N_e = 100,000$ population. Each line represents a different IBD filtering input into the model: random (green; individuals are selected at random), related (orange; 1st-3rd degree relatives are preferentially included), and unrelated (purple; 1st-3rd degree relatives are preferentially excluded). The mean $N_e(t)$ for 25 iterations is plotted and a 95% confidence interval is shaded. In general, in the "related" filtering recent $N_e(t)$ estimates are lower, particularly in IBDNe. HapNe-IBD's smoothing procedure attenuates the choice of filtering, although it converges on an $N_e(t)$ roughly 2x the true $N_e(t)$. HapIBD suffers from oscillations going back as far as 30 generations in the past. The oscillations are more pronounced when relatives are oversampled ("related") or undersampled ("unrelated").

bioRxiv preprint doi: https://doi.org/10.64898/2026.06.15.729614; this version posted June 19, 2026. The copyright holder for this preprint (which was not certified by peer review) is the author/funder, who has granted bioRxiv a license to display the preprint in perpetuity. It is made available under a CC-BY 4.0 International license.

Table 2: RMSE of $\log_{10} N_e$ Ne estimates vs. truth

Demographic scenario	Filtering	IBDNe (0–50 gen)	IBDNe (0–10 gen)	HapNe-IBD (0–50 gen)	HapNe-IBD (0–10 gen)
Out-of-Africa	random	0.126	0.233	0.287	0.292
	random (filtersamples=True)	0.104	0.188		
	related	0.339	0.668	0.258	0.149
	related (filtersamples=True)	0.093	0.175		
	unrelated	0.119	0.224	0.297	0.319
	unrelated (filtersamples=True)	0.119	0.224		
Constant $N_e = 100{,}000$	random	0.073	0.120	0.295	0.298
	random (filtersamples=True)	0.107	0.184		
	related	0.365	0.699	0.273	0.142
	related (filtersamples=True)	0.070	0.134		
	unrelated	0.131	0.239	0.296	0.296
	unrelated (filtersamples=True)	0.131	0.239		
Constant $N_e = 10{,}000$	random	0.031	0.030	0.281	0.292
	random (filtersamples=True)	0.102	0.170		
	related	0.342	0.641	0.259	0.172
	related (filtersamples=True)	0.104	0.172		
	unrelated	0.125	0.215	0.286	0.312
	unrelated (filtersamples=True)	0.125	0.215		
Quebec	random	0.880	1.236	0.769	1.135
	random (filtersamples=True)	0.853	1.250		
	related	0.851	1.369	0.755	1.189
	related (filtersamples=True)	0.860	1.279		
	unrelated	0.890	1.237	0.746	1.141
	unrelated (filtersamples=True)	0.890	1.237		

bioRxiv preprint doi: https://doi.org/10.64898/2026.06.15.729614; this version posted June 19, 2026. The copyright holder for this preprint (which was not certified by peer review) is the author/funder, who has granted bioRxiv a license to display the preprint in perpetuity. It is made available under a CC-BY 4.0 International license.

Table 3: RMSE of $\log_{10} N_e$ Ne estimates vs. truth by sample size (random node sampling only)

Demographic scenario	n	IBDNe (0–50 gen)	IBDNe (0–10 gen)	HapNe-IBD (0–50 gen)	HapNe-IBD (0–10 gen)
Out-of-Africa	250	0.126	0.233	0.287	0.292
	1,000	0.045	0.066	0.268	0.268
Constant $N_e = 10{,}000$	250	0.031	0.030	0.281	0.292
	1,000	0.035	0.036	0.281	0.293
Constant $N_e = 100{,}000$	250	0.073	0.120	0.295	0.298
	1,000	0.018	0.024	0.295	0.297
